# Medico-Chirurgical Transactions, Vol. XVII.—Observations on Tumours

**Published:** 1833-04-01

**Authors:** 


### Medico-Chirurgical Transactions, published by the Medical and Chirurgical Society of London. Volume the Seventeenth. 8vo. 1832.

### Observations on Tumours. By William Lawrence, Esq. F.R.S., &c.

Mr. Lawrence justly remarks that there are few subjects within the circle of his science, more interesting and important to the surgeon, than the history and treatment of tumours. Our information respecting them is yet limited, and although our notions are infinitely more exact and philosophical than they were even a quarter of a century ago, yet our knowledge consists in a consciousness of ignorance, rather than in positive acquisition; in the discrimination of morbid structures, rather than in an acquaintance with the laws which determine their origin, or the means which may accomplish their cure. Mr. Abernethy attempted a philosophical arrangement of tumours. His labours were undoubtedly beneficial, and he probably contributed to the prevalence of rational opinions, but his classification has proved a signal failure.

Mr. Lawrence's paper is on *Tumours.* Mr. Abernethy's classification was of *Tumours.* The term is most unfortunate; it is too comprehensive, and yet not enough so; it comprises growths which are irrelevant to the question, and excludes diseases which must necessarily be involved in its consideration. An organ may be invaded by a morbid alteration of its structure: for instance, by fungus hæmatodes; or the same disease may appear as a new formation, a morbid growth. In a comprehensive, nay, in a merely accurate view of malignant diseases it is impossible to separate altogether morbid growths from morbid alterations of structure. They must be viewed together and yet apart. Mr. Abernethy was hampered by designating his Essay one on Tumours, and as Mr. Lawrence remarks, he was obliged to abandon his definition in the very first page of his work. But the taste of the day is not to split hairs on definitions, which are often no better than logical cobwebs. What the age demands is a careful collection, comparison, and expression of facts.

After making some cursory remarks on the difficulty of separating morbid growths from morbid changes of structure, and the insensible lapse, not only of one into the other, but of various kinds of growths and structures into one another, Mr. Lawrence proceeds to say—

"I nevertheless consider, that Mr. Abernethy's original design of confining the term tumour to new productions ought to be strictly adhered to; and that the distinction between them and changes of structure, although it cannot always be satisfactorily established in our present imperfect knowledge of these affections, is not only useful, but very important both in pathology and treatment. We cannot expect that one and the same explanation will elucidate the origin and growth of an entirely new production, and the alteration of structure in a part; nor can we suppose that common principles of treatment will be applicable in the two cases. Mr. Abernethy, however, makes no distinction between them in his general observations, of which those relating to treatment seem chiefly applicable to changes of structure, while such, as are intended to explain origin and growth, can only be understood as applied to new productions.

Considering that as yet we understand but imperfectly the nature of disease, and the distinctions between such of its forms as are nearly allied, we need not be surprised that it is in many instances difficult, if not impossible, to frame satisfactory medical definitions. Thus, according to Mr. Abernethy's explanation already quoted, the gravid uterus would be a tumour; since it is a swelling caused by a new production, which made no part of the original composition of the body. The French writers seem to me to have done well in classing these growths under the general head of *accidental productions*, as contradistinguished from those which belong to the regular or normal state of the frame; also, in separating them into two divisions, according as they are analogous to the normal tissues, or different from them, *analogues* or *heterologues;* we might say *similar* or *dissimilar.* It will of course be understood, that these run into each other by insensible gradations. We might then define tumours to be accidental productions, either similar to the normal tissues, or dissimilar, developed in the cellular or adipous structures, or in the substance of any organ. These productions are often unattended with any increased magnitude, therefore we must disregard the etymology of the word, and leave the circumstance of enlargement out of our definition." 6.

Mr. Lawrence here falls into a curious inconsistency. He censures Mr. Abernethy for not strictly attending to his own definition of tumour—proposes to use the term in the sense of Mr. Abernethy's definition—then shews that, so used, it is unsatisfactory, and may be applied even to a gravid uterus—lauds the French terms—and winds up by retaining that of tumour, with the proviso that we are to pay no regard whatever to its etymology, and leave the circumstance of enlargement out of our definition.

Mr. Lawrence must really excuse us for remarking, that this is a most lame and halting attempt at scientific precision. Mr. Lawrence next considers the three theories of the formation of tumours—the effusion of blood, its coagulation and organization—the effusion and organization of coagulating lymph—chronic inflammation. It needs no laboured argument to shew their futility. Those who advanced the hypotheses have found it impossible to support them by proof, and the fact is, that we are at present as ignorant of the manner in which new and morbid growths originate, as of the process of natural growth itself. Mr. Lawrence candidly acknowledges that he has no theory to offer on the subject. We take leave, then, of general considerations, which, however they may dazzle or amuse, cannot really instruct, and pass to the consideration of particular facts.

### I. Cellular Tumour.

"It may be observed generally, that the accidental productions constituting tumours frequently correspond in their structure to the parts in which they are produced. Thus we have masses of fat formed in the subcutaneous adipous tissue; and on the other hand, tumours of cellular structure occur in that kind of cellular tissue which does not contain fat. The latter are not so common as the former; and as I have not met with any clear description of this kind of growth, which may be called *cellular* tumour, as the other is termed *adipous*, I shall relate a case of it, observing only that these, like adipous tumours, are not attended with pain or any peculiar symptoms; that they may attain very considerable size; and that they become troublesome or dangerous only in consequence of their bulk." 10.

Case.—*Large Cellular Tumour occupying the Labium Pudendi and Buttock.*

Mr. L. was consulted in 1826 by a lady, aged 28, who told him she had a rupture. A mass, about twice the size of Mr. Lawrence's head, and that is not a bad one, hung from one of the buttocks. Its base was smaller than below, where it expanded into a pendulous mass. The base reached from the coccyx to the left labium, and from the edge of the glutæus magnus to the anus. The tumour was soft, and slightly subdivided into large lobes. The skin was loosely connected to it, and where exposed to friction, was abraded. Some veins of moderate size could be obscurely seen upon the surface. The basis was quite moveable on the subjacent parts, but of uncertain extent towards the front; it was therefore, difficult to determine how far it reached inwards beyond the labium, towards the peritoneum. The health was perfectly good. The complaint had existed for four years, and had not grown fast during the first two. It had commenced at the posterior part of the left labium, and had gradually extended along the buttock and behind the os coccygis.

On Sept. 9th, 1826, Mr. Lawrence removed the tumour. The base of the tumour was readily detached from the parts beneath, a few fibres of the glutæus magnus being exposed. The anterior portion, which advanced into the labium, could not be eradicated ; it passed inwards along the side of the vagina, on which there was an evident drag when this part was pulled; Mr. L. cut it through. The part thus divided was tough, with a compact fibrous texture. There was much faintness from loss of blood. On the 23d, the patient went into the country, the wound being nearly healed.

The tumour consisted of a fleshy mass, undulating on pressure. Its texture on section was nearly uniform, tough and fibrous, and consisting of condensed cellular structure, nearly free from fat, of reddish-grey tint, with some fluid in the interstices.

The lady married, and returned in 1828, far advanced in pregnancy, and with a considerable reproduction of the tumour. It filled the left labium at its back part, and could be felt through the vagina as a kind of cord, ascending towards the pubes. She went through her confinement well, and Mr. L. removed the swelling again on the 1st August, 1828. The anterior prolongation ascended along the left side of the vagina, and seemed to pass under or behind the arch of the pubes. As Mr. L. was putting it on its stretch, it suddenly gave way. He found that it had tapered quite to a point, the end appearing entire, as if the whole morbid growth had come away. The healing went on well, and there has been hitherto no reproduction of the tumour.

In the course of last year Mr. Earle removed at Bartholomew's Hospital a similar tumour, from the left labium pudendi of a female, between twenty and thirty years of age. If we do not much mistake, such tumours are not very uncommon. They grow from the female labium, but, so far as we have seen, they are usually more warty on the surface than in the preceding cases. They attain a large size, and there is usually a free bleeding on their removal. Mr. Lawrence justly remarks that there is this striking difference between the disease under consideration, and the enormous swellings from the male external organs which occur in warm climates—that the latter are the result of more or less violent inflammation.

II. *Tumour occurring in the immediate Vicinity of the Parotid Gland.*

" Mr. Abernethy has described a tumour under the name of *pancreatic sarcoma*, which, he says, 'is made up of irregularly-shaped masses, in colour, texture, and size resembling the larger masses which compose the pancreas. They appear also to be connected with each other, like the portions of that gland, by a fibrous substance of a looser texture.'* He says, that it may occur as a distinct tumour, though it is more frequent as an affection of the female breast ; and he adds, that he has preserved no notes and does not distinctly recollect any case

* " Surgical Observations on Tumours, p. 34."

of a tumour of this structure occurring in a distinct form * The only example that he mentions, with the exception of some instances in the female breast, is that of a man 'with three diseased lymphatic glands, each of the size of a very large plum, situated beneath the basis of the jaw upon the mylohyoideus muscle.† I have seen many instances of tumour approaching in anatomical characters more or less nearly to the description given by Mr. Abernethy of the pancreatic sarcoma, situated close to the parotid gland and near to the angle of the lower jaw; and I have once seen a similar swelling under the basis of the jaw close to the submaxillary gland. The question naturally arises whether the peculiar character of these growths can be referred to their local situation? Whether they derive their resemblance to the structure of the salivary glands from the circumstance of being formed near to them? I think that there is as much analogy in configuration and structure in this case, as we see in any instance between an accidental production and a natural part; and I have not met with a similar tumour in any other situation except in the instance related in Case V., where the new growth occupied the angle of the mouth and side of the lip, and therefore was not far from the parotid.

These swellings have an uneven, knotty, or lobulated surface, as if they were made up of masses more or less distinct. They are firm; sometimes as hard and incompressible as the most dense scirrhous swelling of the breast; sometimes not quite so hard, but still firm. They are loosely connected to the surrounding parts, and therefore easily moveable: this character they retain, however long they may have existed, and are thus distinguished from scirrhus, with which they might be confounded if the mere circumstance of hardness were attended to.

They form and grow without any pain, increasing slowly, so that at the end of five, six, or more years, the tumour may not exceed the size of a walnut. I have not seen it larger than an orange; indeed the complaint causes so much inconvenience and deformity by its bulk, that patients usually submit to an operation before it has attained this size. It is not painful on pressure.

When the tumours of the least firm kind are cut through, they exhibit a very light brownish yellow tint, and an obscurely lobulated arrangement. The colour resembles that of scirrhus, but the texture is less hard and tough; it is softer, and instead of being hard and unyielding, it will break short off. The harder growths are very like scirrhus in density, toughness, and colour: they creak under the scalpel, and sometimes exhibit a few bony particles. Occasionally there is a partial admixture of coagulated blood, in streaks and patches, with the usual texture of the tumour, so as to lead to an apprehension that the disease might be of malignant character.

In some instances the affection has commenced about, or previously to the twentieth year; in others it has appeared later. The swelling has been on the left side in all the cases I have seen; and close on the parotid in all but two, having been in contact with the submaxillary gland in one of these, and situated in the left angle of the mouth and side of the lip in the other." 20.

Mr. L. has always found these tumours innocent, and extirpation by the knife has been successful in every case, excepting one which was operated on by Mr. Macilwain, and which ended fatally from erysipelas. Mr. L. recommends an early operation. The tumour never becomes adherent to the skin nor to the parts beneath, but remains quite loose and moveable throughout. Mr. Lawrence relates five cases. We will give a sketch of them.

---

* Surgical Observations on Tumours, p. 43. † Ib. p. 35.

*Case* 1. The patient was a male, aged 22; the tumour of the size of a large walnut, situated below the left ear, and extending deeply between the mastoid process and jaw. It was dissected out. Partial paralysis of the left side of the face ensued.

*Case* 2. Mary Bray, æt. 34. The tumour was larger than a hen's egg, seated immediately behind the angle and ramus of the jaw. It had commenced seven years previously. Mr. L. removed it. There was profuse bleeding. Both in this and the preceding case, a small slice of the parotid gland was removed with the tumour. The surface of the tumour was tuberculated, and covered by a thin capsule; the substance compact and firm, approaching in colour and consistence to the softer forms of scirrhus, slightly lobulated on its section. The patient left the hospital well on the 22d August.

*Case* 3. A female, aged 45. The tumour as large as a middle-sized walnut, connected by a loose cellular tissue to the parotid; it had existed three years. On section it was not lobulated, resembled cartilage, was very tough, cut with difficulty, and a little bony matter was detected on scraping its cut surface.

*Case* 4. Mr. R., æt. 40, 1826. He had a large swelling over the left side of his lower jaw, irregular, with a prominent knot in front, unattached to the integuments, generally moveable, except at basis, rather elastic. It had appeared eight or ten years previously, and increased slowly without pain or inconvenience.

"An incision was carried over the swelling, from the chin to behind the ear, of which the lobulus was considerably elevated by the tumour: a perpendicular incision along the cheek joined this at right angles. The tumour was quickly denuded on its external surface and sides, but it adhered more firmly at the base, and many arteries were divided in detaching it. I could not separate it in the middle, as it went obviously behind the jaw; I therefore cut it off, and in so doing opened a central cavity, from which fluid escaped. This, I believe, was clear, yellow, and of watery consistence; but, as it mixed with the blood, which was flowing in abundance from numerous arteries, its appearance and nature could not be accurately ascertained. After tying some arteries, I removed from behind the jaw what seemed to be the remainder of the disease; that is, a portion of a cyst with thick sides, which, when fitted to the part previously removed, completed the cavity. There still, however, remained a dark, livid, bloody substance, of spongy texture, going inwards behind the lower jaw. On its front edge the external carotid was beating, completely denuded for about an inch. On examining the margin of this spongy part, I found a thin white cyst, connected by loose cellular tissue, which I dissected and separated cautiously; but I could not get behind it, and I found the internal carotid beating on its posterior margin. The mass broke down readily under pressure with the finger, which passed behind the pharynx, between it and the spine, to the middle of the neck. I broke away what would separate easily; the fragments thus removed were dark reddish or brownish, soft, and friable. After the operation, it was observed that the mouth was drawn to the opposite side, and much distorted.

The tumour was of tolerably firm texture, with a large central cavity; and the sides of the latter were smooth. In colour, which was a light yellowish brown, or light amber, and in general appearance on a section, the texture resembled scirrhus; but it was not so compact or tough. It was invested with a thin but firm white capsule. Towards the cavity, the texture gradually changed into a red and bloody substance. No communication could be ascertained between the interior of the cavity and the spongy prolongation behind the ramus of the jaw." 26.

The structure of the tumour was suspicious. The patient however recovered in about a fortnight, and has remained well to the present time. There is some paralysis of the side of the face.

*Case* 5. A lady, aged 19. The tumour was of the size of a large walnut, and occupied the left side of the upper lip and corner of the mouth. It was quite loose, only covered towards the mouth by the mucous membrane, which was partially everted and exposed to the air. Mr. L. removed it from the inside. The young lady was well in two days.

"Mr. Abernethy seems to doubt whether the swellings in the female breast, which he mentions as illustrations of his pancreatic sarcoma, are new productions, or changes of structure either in the mammary gland or some adjacent lymphatic glands. They obviously belong to the former class, or that of new growths, being generally loose in their situation, surrounded by a distinct capsule, and exhibiting a structure which, although analogous to that of the gland in which they are more or less imbedded, is yet clearly distinguishable from it. They have been well described and delineated by Sir A. Cooper, in his 'Illustrations of the Diseases of the Breast,'* under the name of chronic mammary tumour. They differ from the swellings just described by being much softer and looser in texture, and more distinctly and minutely lobulated throughout: they never exhibit the scirrhous hardness of the others. Again, they are often painful, sometimes causing severe suffering. In this latter respect, as well as in their anatomical characters, they are obviously assimilated to the part in which they are produced." 29.

### III. On Difficulties in the Diagnosis between innocent and malignant Growths.

Mr. Abernethy described a disease under the name of "tuberculated sarcoma," and conceived it "to possess a very malignant nature." Mr. Lawrence has met with a case where such malignancy does not appear to have existed.

*Case.* A tumour appeared spontaneously and increased rather rapidly in the left thigh of a gentleman, aged 27. Four eminent surgeons of London refused, in consultation, to operate. He applied to Sir W. Blizard and Mr. Lawrence.

"We found him with a tumour of elastic feel, undefined in its circumference, about four inches in diameter, with the skin shining and bright red, on the anterior and inner part of the left thigh, a little above the knee. There was a firm indolent swelling, about as large as a hen's egg, imbedded in the soft parts, at the back of the pelvis, and a similar one in the back, near the spine; one as large as a nut over the left eye, and several smaller ones just under the skin in

* Chapter IV. plates 6 and 7.

various parts. All the smaller productions had shewn themselves subsequently to the appearance of the large tumour in the thigh. The patient was almost worn out by pain and want of rest; he was excessively emaciated, with profuse fetid perspiration." 32.

Mr. L. considered the case hopeless, but Sir W. Blizard amputated the thigh two days afterwards, (March, 1819,) and half a year after the first appearance of the swelling. The patient recovered. In 1825 the tumour in the eyebrow was removed. In December, 1828, Mr. L. removed a tumour in the forearm, situate between the ulnar flexor and the bone, and closely adhering to the nerve. The tumour was of firm texture, but not so hard as scirrhus. In December, 1830, the gentleman re-applied with a tumour, the size of a goose's egg, imbedded in the stump. Mr. L. removed it, and in operating found that it was prolonged towards the tuber ischii. The tumour consisted of one of the flexors of the knee, converted into a tough fibrous texture of light brown tint. The wound healed. The pelvic and dorsal tumours remain nearly as they were twelve years ago. Several small subcutaneous knots can be felt in the arms and head, by passing the hand firmly over the surface; but they are less than when the thigh was amputated. There are a few small softish cutaneous growths in the face. The appetite, health, and strength are tolerably good; but there is of late increase of suffering. Strange sensations, sudden dartings and shootings occasioning convulsive movements, and compared to the effects of electricity, are often experienced.

We know not that any remarks can be made on this case, farther than would naturally occur to the well-informed reader from the perusal of the facts.

"Another source of difficulty in deciding on the nature of tumours and the question of operation is found in the circumstance that productions of dissimilar character may be combined in one morbid growth. This may occur in swellings developed in the soft parts, and striking examples of it are sometimes seen in the tumours of bones. From the interior of these organs, more especially of the femur and tibia, there may be produced growths of the medullary kind, which are decidedly malignant, or of the fibrous and osseous description, which are of an innocent character; or of a third kind, in which the two former are blended together. All these productions pass under the unmeaning and vague term of osteo-sarcoma, which it would be better to banish entirely from medical nomenclature. If we speak of fungus hæmatodes, melanosis and cancer of bone; of fibrous and osseous growths, &c. our description may be rendered clear and intelligible, and rational rules of treatment may be laid down." 35.

*Case.* E. W. æt. 30, admitted into Bartholomew's Hospital, June 30th, 1831. There was a tumour nearly as large as her leg, occupying the upper part of the left leg, just below the knee. It was fixed to the tibia—generally firm—in some parts of bony hardness, in others soft and elastic—presenting in front smooth rounded masses, separated by slight depressions—the skin generally healthy, but in front of the head of the tibia thin and livid, and displaying a sloughy ulcerated excavation as large as a half-crown piece. This had been produced by caustic, and from it blood had flowed occasionally, once to the extent of a pint. The tumour had begun in December, 1828. During her pregnancy in 1829 it was stationary; in 1830 it became much larger, and occasionally very painful.

The thigh was amputated on the 2d July. On the 11th August the stump was healed, and the patient left the hospital.

The tumour arose from, and was inseparably connected with, the upper part of the tibia, which seemed to expand into the morbid growth. On section the tumour was seen to consist partly of tough fibrous texture, with bone plentifully deposited in it, partly of medullary substance. It contained numerous cells, filled with transparent yellow fluid, and some containing a small portion of coagulated blood adhering to the surface. Nearly the whole exterior of the swelling, the greater part of the septa between the cells, and the surfaces of the latter were made up of the fibrous and osseous texture; the nucleus of the tumour was composed of the medullary substance. The joint was not penetrated.

"In the cases, which I have seen, of simply medullary or cerebriform growths originating in bones, the progress of the disorder has been much more rapid than in the instance just related; the origin and increase of the morbid production have been attended with pain, generally considerable, and occasionally very severe, with interruption of rest, and more or less disturbance of the circulation and digestive organs. The following case, which I relate in illustration of the differences just mentioned, is interesting in other points of view. I may add that in a man, twenty-two years of age, whose thigh was amputated in St. Bartholomew's Hospital last summer by Mr. Earle, on account of a large medullary tumour growing from the femur, five months only had elapsed from the commencement of the disease, which had been attended with great pain, impaired health, and loss of flesh." 39.

*Case.* Mr. R., in Christmas, 1824, felt a pain in his knee, which was aggravated by exercise, and continued to increase. In February, 1825, he consulted Mr. Lawrence. There were then slight fulness below the knee, slight general swelling, and a little redness about the head of the tibia. Leeches, &c. were prescribed. In March he returned. There was now an elastic tumour between the bones and below the knee, the pulsation of both tibial arteries was suppressed, there was much constitutional disturbance. In the interval between his first and second visit pulsation had been felt in the swelling. The swelling increased rapidly, and the pulsation of the tibial arteries, in the lower part of the limb, returned. The anterior prominence being very soft, a puncture was made; blood only flowed; a bleeding fungus protruded, and there was slight enlargement of the inguinal glands. Amputation was performed. Mr. R. went on well till the night of the 6th day, when he suddenly died from hæmorrhage from the wound.

A medullary tumour occupied the head of the tibia, and had extended forwards and backwards, irregularly between and in the muscles. It had protruded from the bone just at the division of the popliteal artery, and the passage of the anterior tibial through the inter-osseous ligament, which, Mr. L. observes, accounts for the pulsation in the tumour at an early period, the suppression of the tibial pulse when the growth was confined by the fascia of the leg, and its subsequent return when the fascia had given way. An absorbent gland, near the artery, and the inguinal glands, were diseased, as were one or two glands at the side of the pelvis. The end of the femoral artery was quite open. There was a soft medullary tubercle in the thin edge of the liver.

### IV. On the Treatment of Serous Cysts.

"The essential differences that we observe, in the structure of cysts, and the nature of their contents, would lead us to suppose that the same principles of treatment cannot be applicable to all cases of encysted tumours. In some instances the cyst is like the thinnest serous membrane, and contains a clear fluid of watery consistence; in others it is a fibrous structure more or less compact and thick, perhaps with a cuticular or horny lining, or with portions of cartilaginous or osseous texture, and containing either a thick fluid, or from that to the most compact kind of fat or other matter. The fibrous cysts must be either left alone or completely removed by the knife. To irritate them, either by seton, by stimulating injections, or by incision and escharotics, is not only ineffectual as a means of cure, but very dangerous. Measures of the latter kind may be adopted without much risk in the serous cysts. We may make an incision into the swelling and keep it open; inflammation and suppuration of the membrane will ensue, and will lead to permanent obliteration of the cyst. Excision is applicable to the fibrous cysts, which are generally seated in the subcutaneous stratum of adipous texture; while the other mode of proceeding is more convenient in those of serous structure, which are seated more deeply, in the intermuscular cellular tissue, and generally covered, at least in part, by some superficial muscle. These, when neglected, often become so extensive, and are so closely connected to blood-vessels and other important parts, that complete excision would be either extremely difficult or impracticable." 43.

*Case.* A lad, æt. 14, had a swelling on the left side of the neck, which had commenced eight years previously. It was nearly as large as the fist, situated under the sterno-mastoideus, extended from the sternal end of the clavicle to the ear, passed to the side of the trachea, larynx and œsophagus in front, and towards the ligamentum nuchæ behind. It obviously contained fluid.

On Sept. 17th, 1830, Mr. Lawrence made an incision, between two and three inches long, over that part of the swelling behind the sterno-mastoid, and laid bare the cyst, punctured it, and let out a few ounces of light-brownish, watery fluid. The denuded portion of the cyst had a smooth surface, and in it were other smaller cysts, most of them communicating with the general cavity, which was generally smooth. The carotid artery was felt through the thin membrane of the cyst, in the whole length of the neck, as if it had been dissected. Mr. L. introduced four slips of lint, spread with spermaceti cerate, into the cavity, leaving their ends out at the wound, which was lightly covered with a similar dressing. A good deal of tendency to inflammatory action occurred after the operation, and antiphlogistic treatment was necessary. The cavity was obliterated at the beginning of November, when the patient left town. The patient continues well, without a vestige of the former disease.

Mr. Lawrence refers to two cases related by the late M. Delpech. The cysts were very extensive. M. Delpech cured the disease by puncturing the cysts, and introducing bundles of charpie, in such a manner as to come in contact with every part of the internal surface. One case was cured by the 19th day, the other by the 40th.

*Case of Cyst in the Orbit, containing Hydatids.*

C. R. æt. 42, admitted into the London Ophthalmic Infirmary, Jan. 3d,

1820, with protrusion of one eye from the orbit by a tumour, situated between the upper and inner portion of the eyeball and the lid. The upper lid was greatly stretched, the lower everted, the conjunctiva of the globe thickened, the iris motionless, vision gone. The tumour was firm, and apparently fixed to the orbit; it fluctuated obscurely. It had commenced seven years previously. He had applied at the Infirmary when the disease was less advanced, and when only a small firm protuberance could be felt obscurely under the superciliary arch. He then refused to submit to an operation.

Mr. Lawrence made a puncture into the most prominent part of the swelling; a dessert-spoonful of clear watery fluid escaped. Two days afterwards a soft white substance appeared in the puncture; it was removed—it was a hydatid, and a few others escaped when pressure was made on the swelling. The whole collection was cleared out by enlarging the puncture, and injecting water forcibly into the sac. Inflammation and suppuration of the cyst followed, and in about a month the opening closed, the eye returning to its natural situation. In March, a little motion of the iris and slight perception of light had returned.

Professor Delpech relates two cases of serous cyst in the orbit, one containing clear fluid, the other a large hydatid. M. Delpech introduced charpie into both, practice which Mr. Lawrence criticises in the instance of the hydatid, as being unnecessary, and likely to excite too much inflammation.

The profession will feel obliged to Mr. Lawrence for this paper. The publication of the experience of men who have seen much, and particularly of those who, like Mr. Lawrence, are well able to reason on what they see, is always valuable. Perhaps Mr. Lawrence's example may induce others to contribute towards the elucidation of a subject, the importance of which is commensurate with its difficulties.

